# The Impact of Race/Ethnicity on Disparities in Utilization and Outcomes of Neuraxial Anesthesia for Hip and Femoral Shaft Fractures

**DOI:** 10.3390/jcm13143999

**Published:** 2024-07-09

**Authors:** Daniel Qian, Ezekiel Olumuyide, Aakash Keswani, Hung-Mo Lin, Yuxia Ouyang, Samuel DeMaria, Jashvant Poeran, Chang H. Park, Garrett W. Burnett

**Affiliations:** 1 Department of Medical Education, Icahn School of Medicine at Mount Sinai, New York, NY 10029, USA; daniel.qian@icahn.mssm.edu (D.Q.);; 2 Department of Anesthesiology, Perioperative and Pain Medicine, Icahn School of Medicine at Mount Sinai, New York, NY 10029, USAchang.park@mssm.edu (C.H.P.); 3 Department of Anesthesiology, Weill Cornell Medicine, New York, NY 10065, USA; 4 Department of Anesthesiology, Yale School of Medicine, New Haven, CT 06510, USA; 5 Department of Population Health Science and Policy, Icahn School of Medicine at Mount Sinai, New York, NY 10029, USA; 6 Department of Anesthesiology, Critical Care & Pain Management, Hospital for Special Surgery, New York, NY 10021, USA

**Keywords:** health disparities, regional anesthesia, neuraxial anesthesia, trauma, hip fracture, femoral shaft fracture

## Abstract

**Background/Objectives**: The use of neuraxial anesthesia versus general anesthesia for hip fracture surgery remains an active area of research, with recent studies demonstrating mixed findings supporting neuraxial over general anesthesia. The benefits of neuraxial anesthesia have been documented in associated surgeries, including total joint arthroplasty. However, racial disparities in the administration of neuraxial anesthesia have been identified in numerous procedures. We aimed to examine the association of race/ethnicity with neuraxial anesthesia use and the effect of neuraxial anesthesia on length of stay, non-home discharge, 30-day severe adverse events, and rates of readmission among patients undergoing isolated hip and femoral shaft fracture operations. **Methods**: The American College of Surgeons National Quality Improvement Program database was queried for isolated hip or femoral shaft fractures from 2015 to 2019. Stepwise logistic regression was performed to assess the relationship between race/ethnicity and neuraxial anesthesia use. Within each sex–race stratum, neuraxial anesthesia recipients were propensity-matched to general anesthesia recipients in a 1:2 ratio. Logistic regression and negative binomial regression were performed on the propensity-matched cohort. **Results**: A total of 12,004 neuraxial and 64,250 general anesthesia hip and femoral shaft fracture patients were identified. Compared to White patients, Black and Hispanic patients were between 0.64 and 0.61 times less likely to receive neuraxial anesthesia over general anesthesia, respectively (*p* < 0.05). 11,993 patients who received neuraxial anesthesia were propensity matched to 23,946 patients who received general anesthesia. Propensity-matched logistic regressions found that neuraxial anesthesia was associated with decreased length of stay, 30-day severe adverse events, and acute rehab/skilled nursing facility discharge for White patients (*p* < 0.05 for all), but only decreased length of stay in Black and Hispanic patients (*p* = 0.01 and *p* = 0.02, respectively). **Conclusions**: Notable disparities exist in the administration of neuraxial anesthesia for isolated hip and femoral shaft fracture patients. Hispanic and Black race/ethnicity in particular influences provision of neuraxial anesthesia. Further research is required to understand the degree of effect modification and root causes of regional anesthesia access and benefits for this high-volume patient population.

## 1. Introduction

Hip and femoral shaft fractures constitute a significant public health concern, particularly in aging populations, who are at greater risk for these fractures. These fractures are associated with significant morbidity, mortality, and healthcare costs [1,2,3,4]. The high burden of hip and femoral shaft fractures on patients and healthcare expenditure makes it vital to deliver anesthetic care that can improve patient outcomes while minimizing costs.

The use of neuraxial anesthesia (NA) versus general anesthesia (GA) for hip fracture surgery has been extensively studied and remains an active area of research, with some studies favoring NA and others not demonstrating a difference between NA and GA. Studies demonstrating a benefit of NA usage for hip fracture surgery have shown decreased incidence of mortality, blood transfusion, length of stay, cardiac complications, pulmonary complications, and venous thromboembolism when compared to GA [5,6,7]. Most recently, two prospective studies have demonstrated no significant impact of NA over GA regarding mortality, recovery of ambulation at 60 days, or delirium in hip fracture surgery [8,9]. Given these mixed results, and because NA techniques are demonstrably more cost-effective across a variety of orthopedic procedures [10,11,12], further exploration of the value of NA to hip surgery is warranted, particularly at a time when bundled payments in orthopedics (such as the Centers for Medicare & Medicaid Services Comprehensive Care for Joint Replacement model) are becoming more prevalent. 

Racial disparities exist in the management of hip fractures. Black patients who suffer hip fractures are at greater risk for surgical delay, 30-day readmission, reoperation, and 1-year mortality compared to White patients after controlling for patient, hospital, surgeon, and socioeconomic factors [13,14]. Disparities in the provision of NA in hip fracture surgery were recently described and demonstrated a reduced utilization of NA in Black patients when compared to White patients [15]. This finding is consistent with the findings in racial disparities of NA usage in total joint arthroplasty [16,17]. Although the relationship between receipt of NA and race has been previously described, no study has specifically assessed outcomes relating to NA versus GA as it relates to race or ethnicity. 

Our study sought to explore how race/ethnicity impacts anesthesia modality, as well as the effect of that modality on postoperative outcomes in hip and femoral shaft fracture patients. Using propensity-matched cohorts from a national database, we assessed whether race/ethnicity is associated with differences in receipt of NA (as compared to GA) among patients undergoing operative management of hip and femoral shaft fractures, as well as whether race/ethnicity modifies the impact of NA on postoperative complications, readmissions, discharge disposition, and inpatient resource utilization.

## 2. Materials and Methods

### 2.1. Study Design, Data Source, and Study Sample

This retrospective cohort study used data recorded in the American College of Surgeons (ACS) National Quality Improvement Program (NSQIP) dataset, which prospectively collects patient data from over 700 participating institutions [18]. The NSQIP database reports ‘Principal Anesthesia Type’ and, since 2015, has also reported on ‘Additional Anesthesia Technique’ in order to identify combined anesthetic techniques (for example, general and regional anesthesia used concurrently). 

### 2.2. Inclusion and Exclusion Criteria

Patients ≥ 18 years of age who underwent operative treatment for isolated hip or femoral shaft fractures from 2015 to 2019 were identified using common procedural terminology codes. Patients receiving GA or NA (spinal/epidural) based on the ‘principal anesthesia’ and ‘additional anesthesia’ fields were included in the analysis, while all others were removed. Patients with missing data for any variable, including those used in propensity-matching (see below) or postoperative outcomes (see below), were excluded. Patients with procedure codes consistent with multi-system trauma were excluded from the analysis.

### 2.3. Study Variables

Patients were categorized based on whether GA or NA (spinal/epidural) was their primary anesthetic. Descriptive variables for propensity matching included age, demographics, clinical comorbidities, preoperative platelets/INR (International Normalized Ratio), and American Society of Anesthesiology (ASA) physical status classification. Race and ethnicity data collected from the NSQIP database were used to generate a synthetic race/ethnicity variable. Each patient was assigned to one of the following groups: White (non-Hispanic), Black (non-Hispanic), Hispanic, or Other (Asian, Native Hawaiian/Pacific Islander, American Indian/Alaskan Native; non-Hispanic). This classification system is aligned with U.S. Census Bureau standards and has been used in other manuscripts that address health disparities [19]. All patients identifying as Hispanic were categorized into the Hispanic race/ethnicity variable regardless of race identification in order to separately analyze Hispanic patients, especially since these patients are becoming increasingly likely to self-identify their race as Other [20]. Patients who identified as Asian, Native Hawaiian/Pacific Islander, and American Indian/Alaskan Native were grouped into the Other race/ethnicity category, given the relatively lower numbers of these patients in the dataset. 

Outcome variables of interest included length of stay (LOS), non-home discharge (discharge to acute rehabilitation or skilled nursing facility), 30-day unplanned readmission, and 30-day severe adverse events (SAEs). SAEs include death, coma lasting longer than 24 h, ventilator use for greater than 48 h, unplanned intubation, stroke or cerebrovascular accident, thromboembolic event (deep vein thrombosis or pulmonary embolism), infectious complication (superficial or deep surgical-site infection, organ/space infection, or sepsis), cardiac arrest, myocardial infarction, acute renal failure, return to the operating room, and graft/prosthesis/flap failure. 

### 2.4. Propensity-Matching, Statistical Analysis

Study variables were initially compared between the NA and GA cohorts in bivariate analyses using Student’s t-test for normal quantitative variables or a Mann–Whitney U-test for ordinal qualitative or non-normal quantitative variables. Either a chi-square test or Fisher’s exact test was used for nominal qualitative variables and not for discrete quantitative variables. Stepwise logistic regression was performed to assess whether race/ethnicity independently predicted receipt of NA. Propensity scores were then calculated (indicating the propensity to receive neuraxial anesthesia based on all study variables) in a complete multivariable model with type of anesthesia as the outcome. Within each sex–race stratum, using the nearest distance method without replacement, NA patients were propensity-matched to GA patients in a 1:2 ratio. Negative binomial regression for LOS and logistic regression for binary outcomes were performed on the matched cohort to evaluate any effect modification of our race/ethnicity variable. The type of anesthesia, patient race/ethnicity, and the corresponding interaction term were included in the outcome models as covariates. A two-tailed alpha of 0.05 indicated significance for all statistical analyses. All statistical analyses were performed using R version 4.1.2 (R Foundation, Vienna, Austria).

## 3. Results

A total of 12,004 NA and 64,250 GA hip and femoral shaft patients were identified in the initial search query (Table 1). Among all patients (without propensity matching), the neuraxial anesthesia group had a higher mean age (81 vs. 78, *p* < 0.001), a higher percentage of female patients (72% vs. 68%, *p* < 0.001), a lower percentage of patients classified as ASA III and IV (78% vs. 82%, *p* < 0.001), a lower percentage of patients with BMI > 40 (1.1% vs. 2.5%, *p* < 0.001), and a lower percentage of patients who identified as Black or Hispanic as their race/ethnicity (5.9% vs. 10.1% respectively, *p* < 0.001). Those receiving neuraxial anesthesia also had a lower LOS (5.1 days vs. 5.8 days, *p* < 0.001), but higher rates of non-home discharge (77% vs. 76%, *p* < 0.001).

Prior to propensity matching, compared to White patients, Hispanic and Black patients were 0.61 (95% CI 0.55–0.68) and 0.64 (95% CI 0.57–0.72) times less likely to receive neuraxial anesthesia, respectively (*p* < 0.001, Table 2). Patients with a race/ethnicity categorized as Other were 1.79 (95% CI 1.63–1.95; *p* < 0.001) times more likely to receive neuraxial anesthesia compared to White patients. Other independent predictors of neuraxial anesthesia included age (OR 1.03, 95% CI 1.02–1.04) and history of pulmonary disease (OR 1.44, 95% CI 1.35–1.54), while bleeding-causing disorders (OR 0.35, 95% CI 0.32–0.38), ASA III and IV (OR 0.71, 95% CI 0.56–0.91), BMI > 40 (OR 0.56, 95% CI 0.47–0.68), history of chronic heart failure (OR 0.82, 95% CI 0.73–0.93), hypertension (OR 0.92, 95% CI 0.88–0.96) and dependent functional status (OR 0.88, 95% CI 0.83–092) independently resulted in lower likelihoods of neuraxial anesthesia usage (*p* < 0.05 for all). 

**Figure A1:**
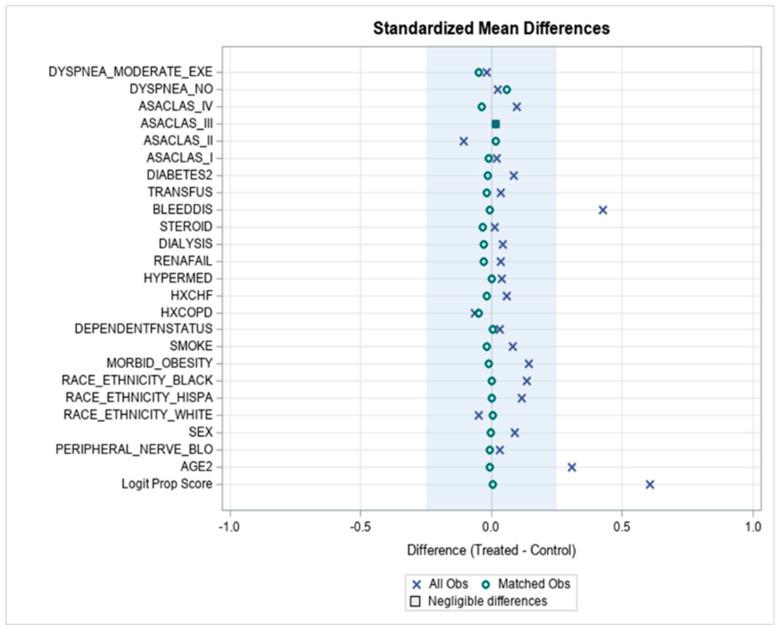
Within each sex–race unique stratum, patients who received neuraxial versus general anesthesia were propensity-matched in a 1:2 ratio using the nearest distance method without replacement. The matched cohort includes 11,993 neuraxial and 23,946 general anesthesia patients.

A propensity score match using a 1:2 ratio of patients receiving neuraxial anesthesia to general anesthesia yielded a cohort of 11,993 patients who received NA and 23,946 patients who received GA. The adequacy of propensity matching was confirmed by a reduction in standardized mean differences in overall propensity scores (Appendix A Figure A1). Negative binomial and logistic regression analyses of propensity-matched patients found neuraxial anesthesia to be associated with decreased LOS (OR 0.93, 95% CI 0.91–0.94), lower likelihood of discharge to an acute rehab or skilled nursing facility (OR 0.89, 95% CI 0.84–0.93), and fewer 30-day severe adverse events (SAEs, OR 0.92, 95% CI 0.86–0.99) for White patients (*p* < 0.05 for all, Table 3). For Black and Hispanic patients, neuraxial anesthesia was only associated with decreased LOS (0.86, 95% CI 0.78–0.96; 0.89, 95% CI 0.81–0.98, *p* < 0.05 for all). For patients identifying as a race/ethnicity in the Other group, neuraxial anesthesia was associated with decreased LOS (OR 0.86, 95% CI 0.81–0.92, *p* < 0.05) and lower likelihood of non-home discharge (0.73%, 95% CI 0.60–0.88, *p* < 0.05).

## 4. Discussion

Our large-scale retrospective study using clinical data from over 76,000 hip and femoral shaft fracture patients found that White patients are more likely to receive neuraxial anesthesia compared to Black and Hispanic patients during operative management of hip and femoral shaft fractures. Our propensity score-matched analysis demonstrated that neuraxial anesthesia is associated with lower LOS, likelihood of non-home discharge, and 30-day SAEs as compared to general anesthesia for White patients, while Black and Hispanic patients only saw a significant reduction in LOS when neuraxial anesthesia was administered.

### 4.1. Race/Ethnicity and Utilization of Neuraxial Anesthesia

Our results demonstrating that non-Hispanic White patients are more likely to receive neuraxial anesthesia for hip/femoral shaft fracture surgery compared to non-White and Hispanic patients are consistent with previous studies in the literature investigating hip fractures, joint arthroplasty procedures, and non-orthopedic procedures, including cesarean section, inguinal hernia repair, and mastectomy [15,17,21,22,23]. Several studies have presented possible explanations for these findings, including the significant roles that English proficiency, preference for English as the spoken language of care, and patient lack of information and awareness of options can play in the anesthetic approach decision-making process [24,25]. Additionally, non-White patients may be more likely to select conservative anesthetic approaches due to limited trust in health systems and providers [26,27]. The traumatic nature of these fractures and the non-elective timing of these particular surgeries further exacerbate this issue, as patients may lack sufficient time to develop trust-based relationships with their providers [28]. These factors present potential barriers to neuraxial anesthesia and can complicate conversations between provider and patient regarding the anesthetic plan of care. Other authors have identified provider-specific factors driving access to neuraxial anesthesia; Zhong et al. found that significant disparities in receipt of neuraxial anesthesia by race in total knee arthroplasty patients disappeared in models that controlled for provider characteristics (e.g., hospital location, teaching hospital, hospital size, region) [17]. This suggests that while differential access to neuraxial anesthesia exists on a population level, modifiable hospital-specific factors such as protocol standardization can guide potential targeted interventions to address these differences in access. Future studies should investigate provider- and hospital-level factors as drivers of neuraxial anesthesia access in patients with hip and femoral shaft fractures undergoing operative management. Although, as with most large database studies, no root causality can be inferred from our results, it is clear that racial/ethnic differences exist in the provision of neuraxial anesthesia, the etiology is likely multifactorial, and physicians can proactively engage in shared decision-making and focused communication techniques in order to begin reducing these disparities [29].

It is worth noting that patients categorized in the Other race/ethnicity group, which included patients identifying as Asian, Pacific Islander, and Native American/Alaskan Native, were more likely to receive neuraxial anesthesia for hip and femoral shaft fractures than White patients. This finding is noteworthy because other studies on access to neuraxial anesthesia have demonstrated that these racial/ethnic groups tend to be less likely than both White and Black patients to receive neuraxial anesthesia, albeit for non-orthopedic procedures [21,22,23]. One possible explanation for this discrepancy relates to the aforementioned concept of trust in health systems. Patients identifying as Asian have previously been shown to report significantly greater trust in the medical community [30]. Given that our Other race/ethnicity cohort was composed mostly of Asian patients, this positive predisposition may be driving up the group’s likelihood of receiving neuraxial anesthesia in our analysis. This discrepancy indicates that studies should work to discern whether these juxtaposed results can be tied to procedure type (e.g., orthopedic vs. non-orthopedic), as well as to disaggregate the racial/ethnic groups currently classified into Other through sample sizes that would allow for more granular multivariable analysis.

Our findings indicate that certain comorbid conditions are also associated with different likelihoods of NA administration, and the literature suggests that non-White patients exhibit disproportionately higher comorbidity burdens [31]. However, this association should not be interpreted as a rationale for the lower odds of Black and Hispanic patients receiving neuraxial anesthesia, given that not all comorbidities in this analysis are independently associated with neuraxial anesthesia access, and a history of pulmonary disease is associated with a higher likelihood of neuraxial anesthesia provision. Additionally, more broadly, the most consequential contraindications for regional anesthesia include coagulopathies, neurologic deficits, acute infection at injection site, and lack of patient consent. Apart from the decreased likelihood of NA usage in patients with bleeding disorders (OR 0.35, 95% CI 0.32–0.38), none of the other conditions analyzed in our study are incompatible with neuraxial anesthesia. The impact of these covariates should be evaluated across all patient groups in future investigations and is beyond the scope of this study. 

### 4.2. Race/Ethnicity and Outcomes/Resource Utilization

Out of the four outcome and resource utilization metrics studied (LOS, non-home discharge, 30-day unplanned readmission, 30-day SAEs), White patients experienced significant beneficial effect modification of neuraxial anesthesia in three of them (LOS, non-home discharge, 30-day SAEs). In contrast, Black and Hispanic patients only experienced significant effect modification by NA in one metric (LOS), and Other race/ethnicity patients saw significant modification in two (LOS, non-home discharge). It should be noted, however, that Black patients saw a non-significant reduction in non-home discharge, 30-day readmission, and 30-day SAEs, and Hispanic patients also saw a non-significant reduction in 30-day SAEs. In addition, contrary to investigator expectations, the degree of reduction across each of these outcome metrics was greater than those for White patients, although again, the results are not significant.

In other words, all groups experienced a reduction in inpatient resource utilization when neuraxial anesthesia was applied, which is in alignment with the public health goal of improving value through cost reduction. Additionally, NA supports improved postoperative outcomes for White patients in hip and femoral shaft fracture procedures, thereby improving quality. While we do not have enough evidence to support similar claims for Black and Hispanic patients, the optimistic directionality of our results can guide future research efforts. As demonstrated by the White patient group, the potential for benefit from neuraxial anesthesia in this patient population exists; improving access across all groups, as discussed earlier, may provide more robust data that would allow us to more comprehensively evaluate these quality improvement outcomes.

Our study further expands upon the results from the REGAIN trial by introducing a race/ethnicity component and exploring how this demographic variable corresponds to differences in anesthetic outcomes [8]. While REGAIN did not focus on NA usage, several secondary and exploratory outcomes overlapped with data we examined from the ACS-NSQIP database, and it is worth noting how these dependent variables are different. With respect to SAEs, the REGAIN trial followed in-hospital complications (and found lower rates of death during hospitalization, AKI, and CCU admission among spinal anesthesia patients compared to general), while we measured all 30-day SAEs. Our primary outcome variable of non-home discharge was an exploratory outcome in the REGAIN trial, where spinal anesthesia was similarly associated with a lower rate of non-home discharge. And while both studies included a duration outcome, our total LOS measure and REGAIN’s time from randomization to discharge represent different starting points. Our results align with Neuman et al.’s previous findings that NA in hip surgery is associated with a modest LOS reduction and our remaining primary outcome measures are distinct from those previously explored [32].

Our study is the first to specifically note a correlation between race/ethnicity and the degree of benefit experienced from NA in this patient population. It should be noted that race is a social construct, as opposed to biological; as such, race can serve as a proxy for the environment in which a patient lives, their cultural traditions and attitudes, and their propensity to engage in health-improving or preserving behaviors—all of which would have an effect on LOS and non-home discharge [33]. In identifying these trends in outcomes from NA in traumatic hip and femoral shaft fracture, our goal is to motivate investigators to uncover patient, provider, and societal root causes, as well as strategies to harness the potential of NA to achieve health equity. 

### 4.3. Limitations

There are several important limitations to our analysis. With respect to health disparities, the NSQIP database includes only sex, race, and ethnicity without access to additional factors to assess social deprivation, including income, insurance status, ZIP code, level of education, substance abuse history, homelessness, HIV/AIDS status, and mental health status, among other comorbidities. This analysis additionally lacks provider- and hospital-level factors that have been shown to influence access to neuraxial anesthesia, such as physician/hospital quality rating, hospital annual hip fracture volume, total case volume, hospital bed size, percentage of total inpatient cases by hospital that receive neuraxial anesthesia, hospital payer mix, percentage of anesthesiologists with regional anesthesia fellowship training, and academic versus non-academic medical center status. Given the parsimonious nature of our model, it would be valuable for future studies to evaluate these and other confounding factors that could potentially explain our outcomes. Another limitation of the NSQIP database is the inability to capture SAEs and readmissions in locations other than the hospital where the index surgery was performed. Other metrics that future studies can use to better understand the nature of NA health disparities include more granular anesthesia data (including agents and techniques used for GA/NA), extending the time period for collection of adverse events to 6 months or 1 year, and focusing on patient-centered outcome metrics (e.g., patient-reported outcomes and postoperative functional status). Finally, it should be noted that the NSQIP database is populated manually, and the literature reports potential inaccuracies specifically pertaining to hip fracture CPT codes and associated 30-day complications [34]; while the limitations of NSQIP and other databases that rely on administrative data should be acknowledged, they remain valuable population-level resources that will help us to begin answering questions related to health equity. 

## 5. Conclusions

This retrospective study using ACS-NSQIP data from 2015–2019 demonstrated evidence that non-Hispanic White patients are more likely to receive neuraxial anesthesia than non-Hispanic Black and Hispanic hip and femoral shaft fracture patients. These disparities are important because, as demonstrated in our outcomes analysis, neuraxial anesthesia offers a viable avenue to lower inpatient resource utilization based on hospital length of stay. Further research is needed to continue evaluating the potential postoperative outcome benefits of neuraxial anesthesia among various racial and ethnic groups, especially since we have seen significant lower likelihoods of non-home discharge and 30-day SAEs with White patients. While the underlying reasons that explain these differences between racial/ethnic groups have not been fully elucidated, it is clear that patient, provider, and societal factors play important roles in the persistence and evolution of these health disparities in this growing, high-burden patient population.

## Figures and Tables

**Table 1:** Bivariate analysis of patient/procedure characteristics among general versus neuraxial anesthesia patients prior to propensity matching (BMI = Body Mass Index, ASA = American Society of Anesthesiology physical status classification, INR = International Normalized Ratio, LOS = Length of Stay).

	General Anesthesia 64,520 (100%)	Neuraxial Anesthesia 12,004 (100%)	*p*-Value
Age (mean)	78 (SD 12)	81 (SD 9)	**<0.001**
Race/ethnicity			**<0.001**
White	55,927 (87%)	10,596 (88%)	
Black	3174 (4.9%)	327 (2.7%)	
Hispanic	3345 (5.2%)	380 (3.2%)	
Other	2074 (3.2%)	701 (5.8%)	
Male gender	20,347 (32%)	3310 (28%)	**<0.001**
ASA III and IV	53,174 (83%)	9398 (78%)	**<0.001**
Dependent functional status	13,340 (21%)	2322 (19%)	**0.003**
BMI > 40	1625 (2.5%)	127 (1.1%)	**<0.001**
History of smoking	8617 (13%)	1298 (11%)	**<0.001**
History of diabetes	12,578 (20%)	1970 (16%)	**<0.001**
History of pulmonary disease	6868 (11%)	1541 (13%)	**<0.001**
History of chronic heart failure	2430 (3.7%)	335 (2.8%)	**<0.001**
Hypertension	43,281 (67%)	7835 (65%)	**<0.001**
History of renal disease	439 (0.7%)	53 (0.4%)	**0.002**
Steroids for chronic condition	3586 (5.6%)	637 (5.3%)	0.28
Bleeding-causing disorders	11,795 (18%)	871 (7.3%)	**<0.001**
Pre-operative blood transfusion	2797 (4.3%)	443 (3.7%)	**0.001**
Laboratory results within 90 days pre-op. (%)			
Low platelets (<100,000/mcL)	2655 (4.1%)	232 (1.9%)	**<0.001**
High INR (>1.4)	58,766 (91%)	284 (2.4%)	**<0.001**
Peripheral nerve block	2806 (4.4%)	447 (3.7%)	**0.002**
Operative time (mean)	69 (SD 44)	66 (SD 39)	**<0.001**
Hospital LOS (mean)	5.8 (SD 4.2)	5.1 (SD 3.6)	**<0.001**
Discharge Disposition			**<0.001**
Acute rehab facility	14,167 (22%)	2666 (21%)	
Skilled nursing facility	34,658 (54%)	7135 (56%)	
Home/other	15,695 (24%)	2924 (23%)	

Bold values denote statistical significance at the *p* < 0.05 level.

**Table 2:** Stepwise logistic regression of factors associated with neuraxial anesthesia use in non-propensity-matched hip fracture patients.

Predictive Factors	Odds Ratio (95% CI)	*p*-Value
Age	1.03 (1.02–1.04)	**<0.001**
Operative time	-	-
Black Race/ethnicity ^1^	0.64 (0.57–0.72)	**<0.001**
Hispanic Race/ethnicity ^1^	0.61 (0.55–0.68)	**<0.001**
Other Race/ethnicity ^1^	1.79 (1.63–1.95)	**<0.001**
Male gender	-	-
Dependent functional status	0.88 (0.83–0.92)	**<0.001**
BMI > 40	0.56 (0.47–0.68)	**<0.001**
History of smoking	-	-
History of diabetes	-	-
History of pulmonary disease	1.44 (1.35–1.54)	**<0.001**
History of chronic heart failure	0.82 (0.73–0.93)	**0.001**
Hypertension	0.92 (0.88–0.96)	**0.001**
History of renal disease	-	-
Steroids for chronic condition	-	-
Bleeding-causing disorders	0.35 (0.32–0.38)	**<0.001**
Pre-operative blood transfusion	-	-
ASA III and IV	0.71 (0.56–0.91)	**<0.001**
Laboratory results within 90 days prep. (%)	-	-
Low platelets (<100,000/mcL)	-	-
High INR (>1.4)	-	-

^1^ as compared to White race/ethnicity reference. Bold values denote statistical significance at the *p* < 0.05 level.

**Table 3:** Propensity score-matched logistic regressions assessing effect modification by race/ethnicity of neuraxial anesthesia on postoperative outcomes and resource utilization (SNF = skilled nursing facility).

	Length of Stay		Acute Rehab/SNF Discharge		30-Day Unplanned Readmission		30-Day Adverse Events	
	Mean Ratio (95% CI) ^1^	*p*-Value ^2^	Mean Ratio (95% CI) ^1^	*p*-Value ^2^	Mean ratio (95% CI) ^1^	*p*-Value ^2^	Mean ratio (95% CI) ^1^	*p*-Value ^2^
Effect modification of Race/ethnicity		**0.01**		**0.01**		0.28		**0.68**
White	0.93 (0.91–0.94)	*	0.89 (0.84–0.93)	*	1.04 (0.96–1.14)		0.92 (0.86–0.99)	*
Black	0.86 (0.78–0.96)	*	0.85 (0.64–1.13)		0.79 (0.50–1.25)		0.74 (0.50–1.10)	
Hispanic	0.89 (0.81–0.98)	*	1.29 (0.97–1.72)		1.01 (0.68–1.46)		0.86 (0.57–1.31)	
Other	0.86 (0.81–0.93)	*	0.73 (0.60–0.88)	*	1.36 (0.97–1.89)		0.99 (0.74–1.36)	

^1^ as compared to general anesthesia use as reference. ^2^ *p* value for the race/ethnicity interaction with anesthesia type. * indicates a significant odds ratio for effect of neuraxial anesthesia on resource utilization and postoperative outcomes. Bold values denote statistical significance at the *p* < 0.05 level.

## Data Availability

The original data presented in the study are openly available in the National Surgical Quality Improvement Program from the American College of Surgeons at https://www.facs.org/quality-programs/data-and-registries/acs-nsqip/, accessed on 5 January 2022.
